# Assessing knowledge, attitude, and practices of veterinarians towards antimicrobial use and stewardship as drivers of inappropriate use in Abuja, Nigeria

**DOI:** 10.1186/s42522-021-00058-3

**Published:** 2021-12-20

**Authors:** Mabel Kamweli Aworh, Jacob Kwada Paghi Kwaga, Emmanuel Chukwudi Okolocha

**Affiliations:** 1 Department of Veterinary and Pest Control Services, Federal Ministry of Agriculture and Rural Development, New Secretariat, Area 11, Garki, Abuja, Nigeria; 2 Nigeria Field Epidemiology and Laboratory Training Programme, Abuja, Nigeria; 3 Department of Veterinary Public Health and Preventive Medicine, Ahmadu Bello University, Zaria, Nigeria

**Keywords:** Knowledge Assessment, Prescribing Practices, Antimicrobial Resistance, Antimicrobial Stewardship, Antimicrobial Use, Nigeria

## Abstract

**Introduction:**

Antimicrobial resistance (AMR) has recently gained worldwide recognition, as the emergence of multi-drug resistant organisms has led to increased mortality and economic burden. This study aimed to assess knowledge, attitudes, and practices of veterinarians towards rational antimicrobial prescribing and identify factors influencing use.

**Methods:**

We interviewed veterinary doctors in Abuja, Nigeria using a 50-point questionnaire distributed via WhatsApp mobile application. The questionnaire inquired about their experiences, knowledge, attitudes, and practices towards AMR and stewardship. We analyzed the data by calculating frequencies and proportions.

**Results:**

Of 220 registered veterinarians, 144 (65.5%) participated in the survey. Most (52.8%) were within the age group 30 - 39 years; males (72.2%), with a Master’s degree (42.4%) and worked in public service (44.4%). Three-quarters (75.7%) had good knowledge of antimicrobials; 47.2% had received training on stewardship while 88.9% reported that they believed that overuse of antimicrobials was the major contributory factor towards AMR. Antimicrobial stewardship regulations are important in veterinary practice. Veterinarians were aware of the occurrence of resistant pathogens and agreed that restricting antimicrobial use in animal health care was necessary to reduce AMR.

**Conclusion:**

Most respondents referred to the veterinary formulary (VF) when in doubt of the appropriate antimicrobial agent to administer. We recommend that the VF be updated following the WHO list of critically-important-antimicrobials (CIA) and veterinarians educated not to use these CIAs in the treatment of food animals.

**Supplementary Information:**

The online version contains supplementary material available at 10.1186/s42522-021-00058-3.

## Introduction

Antimicrobial resistance (AMR) in these present times has become a big challenge to public health worldwide, because of the emergence of superbugs “*which are strains of bacteria that have become resistant to antibiotic drugs*” hence resulting in higher mortality and economic hardships [1]. Sub-Saharan African countries are also faced with the challenges arising from AMR. This global health problem is facing both medical and veterinary healthcare professionals, hence it requires a ‘One-Health’ approach to provide effective response [2]. Antimicrobials are used in food-producing animals for prevention, growth promotion, or treatment of animal diseases. Evidence has shown that a linkage exists between antimicrobial use in animals and the resultant development of AMR [3]. Documentary evidence has shown that abuse of antimicrobials when treating human and animal illnesses may be the reason for the selective rise in certain resistant populations [4]. In Nigeria, AMR is becoming an issue of concern by the increase in prevalence of multidrug resistant foodborne pathogens in food producing animals as evident in many studies [5–8]. Furthermore, evidence shows that the prevalence of hospital acquired infections in Nigeria ranged from 6.3 – 15% [9–11].

The World Health Assembly (WHA) passed resolution 68.7 in May 2015 requesting that member states participate in an integrated global programme for surveillance of AMR and adopt a country-specific action plan according to the Global Action Plan on AMR. As part of Nigeria’s AMR prevention and control initiative, a situation analysis of AMR was conducted and a National Action Plan (NAP) on its implementation was developed in 2017. Gap analysis identified a lack of antimicrobial stewardship (AMS) programmes in both the private and public sectors. Findings from this situation analysis also revealed that the animal health sector lacked AMS programmes in veterinary hospitals, clinics, and farms [12]. Consequent upon this, one of the focus areas that the Nigerian AMR NAP is promoting, is rational access to antibiotics and AMS [13]. AMS programmes encourage the use of treatment guidelines and ensure prudent use of antimicrobials in providing health care to humans and animals [13]. AMS promotes optimal prescribing and dispensing of antimicrobials in humans and animals hence preserving important antibiotics for urgent clinical needs [2, 13]. In the context of addressing AMR using the One Health approach, the veterinary professionals must imbibe the concept of AMS which originated within the human healthcare system [4].

AMS programmes are financially rewarding for hospitals engaged in such programmes. However, a patient’s outcome is associated with antibiotic costs in performing such programmes [14]. AMS programmes are aimed at ensuring that the correct antimicrobials are selected, along with appropriate dosing, correct route of administration, and duration of use to ensure better outcomes and lower costs for the patients [14, 15]. AMS practices are encouraged among veterinarians to reduce indiscriminate use of antimicrobials as well as improve antimicrobial use in animal health care delivery.

In Nigeria, AMR governance structures have been established across human health, animal health, and environmental health under the umbrella of One Health. However, only the human health component has made tremendous efforts in establishing AMS programmes across hospitals in Nigeria [13]. The concept of AMS is relatively new in veterinary practice in Nigeria where there is unregulated access to antimicrobials in the provision of health care to animals especially food animals.

The veterinarian requires in-depth knowledge of veterinary medicine to make a clinical diagnosis and make the right choice of antimicrobials; therefore knowledge is the basis of these AMS programmes [16]. Behavioural change which can be difficult has been improved among physicians because of increased awareness hence they have responded positively to educational interventions and have become good antibiotic stewards [14]. Knowledge, attitude, and practice (KAP) studies are targeted at discovering and understanding a peculiar population's knowledge and approach towards a particular topic of interest hence conducting such studies bring to the fore existing knowledge gaps. Some KAP studies on AMR have been conducted among relevant stakeholders in Nigeria which identified gaps in AMS awareness [17–21]. Studies carried out among Nigerian veterinary students reported that 60% had an unsatisfactory level of knowledge of AMR while only about 7% knew the meaning of antimicrobial stewardship [18, 19]. To our knowledge, there is no currently available citable literature on knowledge, attitude, and practices of veterinarians towards antimicrobial use and stewardship in Abuja.

To this end, we assessed the knowledge, attitude, and practices of veterinarians towards antimicrobial use and AMS in Abuja, Nigeria as well as identify factors that influenced antibiotic prescribing practices of veterinarians. Findings from this study will provide baseline data required to inform interventions aimed at promoting AMS among veterinarians and ultimately guide policy formulation in the animal health sector.

## Methods

### Study Design

This cross-sectional survey was conducted among 144 veterinary doctors practicing in Abuja, Nigeria using a pretested 50-point structured questionnaire distributed by sending a web link via the WhatsApp mobile phone application and email. The questionnaire inquired about the veterinarians’ experiences, knowledge, attitudes, and practices towards antimicrobial resistance and antimicrobial stewardship. The questionnaire also included a survey of the antimicrobials sold by veterinary outfits.

### Data Management and Analyses

We reviewed completed questionnaires upon receipt online to ensure that they were correctly filled by the respondents. Data collected were cleaned and analyzed using Epi info version 7. We analyzed the data to obtain indicators of the veterinarians’ knowledge, attitude, and current practices on AMR and AMS. Knowledge and attitude questions were isolated and scored. A knowledge score was calculated for each respondent based on 12 knowledge questions. We assigned scores based on the question’s likert scale: with scores ranging from One (1) for least confident to Four (4) for most confident. For other questions, One (1) mark was given for every correct response and zero (0) for an incorrect response. Responses of “Do not know” were counted as incorrect, and no points were given. The total knowledge score was the sum of all the correct answers provided by the respondent. Mean knowledge score (%) was calculated and divided into three categories: poor (<60%), average (60-80%), and good (>80%) level. We applied the same criteria used for knowledge questions to assign scores for questions on attitude hence mean attitude score (%) was calculated and divided into three categories: poor (<60%), average (60-80%), and good (>80%) level. Further data analyses were conducted using Chi-square and Fisher’s exact test to determine the association between categorical variables. Results were presented using the odds ratio at a 95% confidence interval. Test results were considered significant if the p-value is < 0.05. Factors found to be significant in the bivariate analysis were included in the multivariate analyses.

## Results

### Demographic Characteristics of Study participants

**Fig. 1:**
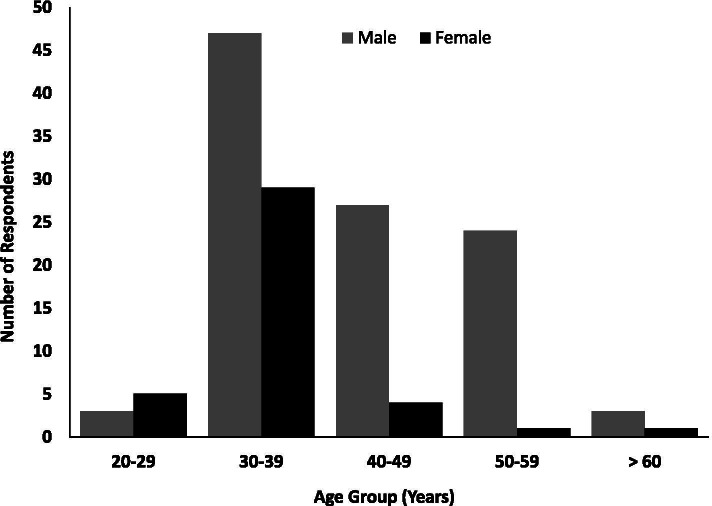
Age and Sex distribution of the respondents in Abuja, 2020. Most of the veterinarians working in Abuja who participated in this survey both male and female were within the age group 30 – 39 years. The least number of respondents for both genders were above 60 years of age

Veterinarians in Abuja are representative of veterinarians in the North-Central part of Nigeria. Of 144 veterinarians who participated in this survey, 104 (72.2%) were men and 40 (27.8%) were women. Most of the respondents 76 (58.3%) were within the age group 30 - 39 years. Some of the respondents 69 (47.9%) had a Master’s degree; 65 (45.1%) worked with the government and 108 (75%) mostly within Abuja Municipal Area Council (AMAC). Table 1 shows the demographic characteristics of the respondents**.** Figure 1 shows the age and sex distribution of the respondents. A majority (77.8%) of the respondents graduated from veterinary school after the year 2000 and obtained a Doctor of Veterinary Medicine (D.V.M) degree (Table 1).

**Table 1:** Demographic Characteristics of Veterinarians in Abuja, Nigeria, 2020

Characteristics	Frequency N=144	Percent (%)
**Qualification:**		
DVM	59	41.0
MSc/MPH	69	47.9
PhD	7	4.9
Others	9	6.3
**Field of Practice:**		
Government (Public Service)	65	45.1
Academia	19	13.2
Private practice	49	34.0
Vet Pharmacy	4	2.8
Others	7	4.9
**Location:**		
AMAC	108	75.0
Gwagwalada	14	9.7
Kuje	10	6.9
Bwari	10	6.9
Kwali	1	0.7
Abaji	1	0.7
**Year of Graduation (DVM):**		
Before year 2000	32	22.2
After year 2000	112	77.8

### Knowledge of AMR and Stewardship

There were twelve knowledge questions assigned scores based on the question’s likert scale and one mark for each correct answer. The mean knowledge score was 66% with a standard deviation of 14.1 out of a maximum score of 100%. Of the 144 veterinarians who participated in this survey, only 18.1% (n = 26) had good knowledge of AMR and stewardship. Less than half of the respondents (48.6%, n= 70) had average knowledge of the subject while 33.3% (n= 48) had poor knowledge (Table 2).

**Table 2:** Level of Knowledge demonstrated by respondents on AMR and AMS, Abuja

**Level of Knowledge**		**Frequency**	**%**
Overall Knowledge			
Mean Knowledge Score (%)		66.3 + 14.1	
Good Knowledge		26	18.1
Average Knowledge		70	48.6
Poor Knowledge		48	33.3
**Specific Questions**	**Responses**	**Frequency**	**%**
*Antimicrobial resistance is an issue of concern in Nigeria*	Strongly Agree	78	54.2
	Agree	50	34.7
	Strongly Disagree	16	11.1
*Antimicrobials are effective in treating acute viral infections*	Yes	31	21.5
	No	113	78.5
*Are there risks associated with the irrational use of antimicrobials?*	Yes	117	81.3
	No	27	18.8
*Do you think restricting usage is necessary to reduce AMR?*	Yes	113	78.5
	No	31	21.5
*Do you believe that missing doses of antimicrobial agents contribute to AMR?*	Yes	128	88.9
	No	16	11.1
*Are there possibilities that new classes of antimicrobials will be developed in the next 5 to 10 years?*	Yes	89	61.8
	No	11	7.6
	Don’t know	44	30.6
*Are you aware that you may be contributing to AMR in your veterinary practice?*	Yes	91	63.2
	No	12	8.3
	May be	41	28.5
*Have you attended any workshops or training on AMR or AMS?*	Yes	68	47.2
	No	76	52.3
*Would you like more education/training on AMR or AMS?*	Yes	134	93.1
	No	1	0.7
	May be	9	6.3

***Note:*** *Scoring assumptions: poor (< 60%), moderate (60 – 80%), and good (> 80%)*

Most 88.9% (n=128) of the respondents agreed that AMR was an issue of concern in Nigeria while 78.5% (n=113) agreed that antimicrobial agents were not the appropriate medication for treating acute viral infections. Regarding factors that contributed to AMR in Nigeria, 88.9% (n=128) declared that the overuse of antimicrobials without prescription was the most significant contributory factor. This was followed by non-compliance of clients/farmers with the prescribed treatment 75.7% (n=109); use of antimicrobials as growth promoters in animals 65.3% (n=94); farmers’ pressure on veterinarians for antimicrobial prescription 48.6% (n=70); errors in veterinary prescription – dose, duration of use and choice 47.9% (n=69). Overuse of antimicrobials by prescription 42.4% (n=61); inadequate hygiene and biosecurity measures 34% (n=49); lack of vaccinations 21.5% (n=31) and lack of new antimicrobial drugs 14.6% (n=21).

Concerning prescribing antimicrobials for use in animals, 63.2% (n=91) of the respondents were confident in making an accurate diagnosis of infection; 35.4% (n=51) were confident about when to take a decision not to prescribe antimicrobials especially when not sure of the diagnosis. Less than half 47.2% (n=68) were confident about selecting the correct antimicrobial agent; 41.7% (n=60) were confident about selecting the correct dosage of antimicrobial prescribed while 36.1% (n=52) were confident about when to stop using the antimicrobial agent.

A majority of the respondents, 81.3% (n=117) agreed that there were certain risks associated with the irrational use of antimicrobials in treating food-producing animals; however, 76.4% (110) of them were able to correctly identify the possible risks associated with irrational use of antimicrobials. Many of the respondents, 78.5% (n=113) agreed that restricting access to antimicrobials for use in veterinary care was necessary to reduce the menace of AMR in Nigeria. Most 88.9% (n=128) of the respondents agreed that failure to administer one or two doses of antimicrobial agents to a patient during treatment regimen also contributed to the development of AMR; however, 76.4% (n=110) of them correctly listed three important resistant bacteria identified for AMR surveillance in Nigeria namely *E. coli, Salmonella, Staphylococcus aureus*. Less than half, 47.2% (n=68) of the respondents declared that they had attended a workshop or training on AMR and stewardship.

### Attitudes of veterinarians towards rational prescription and use of antimicrobials

**Fig. 2:**
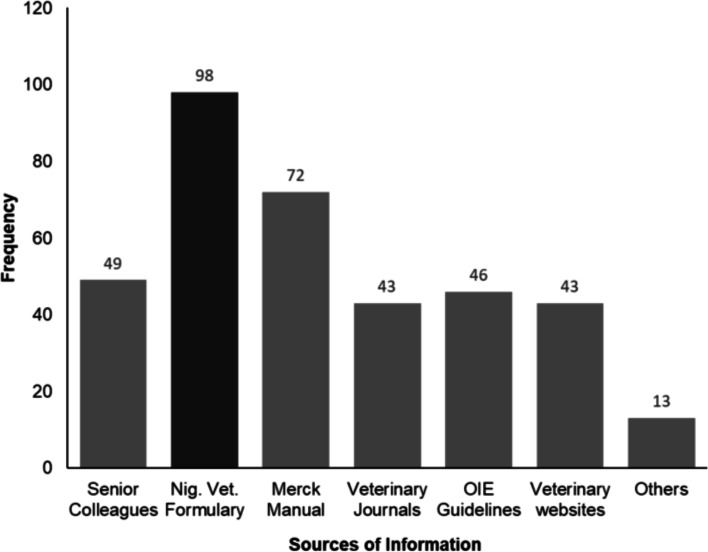
Sources of information that guides Veterinarians on appropriate antimicrobial use in Abuja, 2020

For this study, respondents were considered to have good attitude when their overall attitude score was over 80%. The mean attitude score was 73.4% with a standard deviation of 20.5 out of a maximum score of 100%. Thirty-nine respondents (27.1%) demonstrated a poor attitude regarding AMS while 68 (47.2%) of the respondents displayed moderate attitude. Only 37 (25.7%) of the respondents had a good attitude regarding AMS. Regarding AMS, 56.9% (n=82) of the respondents declared that antimicrobials are safe drugs that can be commonly prescribed. Most 82.6% (n=119) of the respondents agreed that prescribing antimicrobials to healthy animals as a form of prophylaxis may harm the health of these animals. Regarding sources of information, 67.1% (n=94) of veterinarians referred to the VF for information on appropriate usage of antimicrobials while 49.3% (n=69) consulted the Veterinary Merck Manual **(**Fig. 2**)**.

Each bar represents the various sources of information regarding antimicrobial use. Most (67.1%) veterinarians working in Abuja who participated in this survey usually refer to the Veterinary Formulary

Regarding important strategies to combat AMR, 92.9% (n=130) of the respondents suggested that educational campaigns were the most appropriate strategy to adopt; 77.1% (n=108) of respondents suggested that the use of treatment guidelines was appropriate; 78.6% (n=110) of respondents suggested that better control of antimicrobial sales were appropriate. Some 73.6% (n=103) of respondents suggested that reducing antimicrobial use in animals was appropriate; 72.1% (n=101) of respondents suggested that improved biosecurity in farms, clinics, and hospitals was appropriate while 41.4% (n=58) suggested vaccination campaigns were appropriate.

### Current antimicrobial prescribing practices among veterinarians

Over 92% (n=133, 92.4%) of respondents possessed the annual veterinary practicing license issued by the Veterinary Council of Nigeria. More than two-thirds (n=97, 67.4%) of the respondents prescribed antimicrobial agents to their clients for use in animals. Among those who indicated that they prescribed antimicrobials for use in animals, 11 (12.6%) prescribed an average of 2 – 3 doses of antimicrobials daily while seven (8%) usually prescribed over five (5) doses of antimicrobial agents daily. Slightly over two-thirds (n=97, 67.4%) prescribed doses of antimicrobials based on observation and experience. However, less than half (n=45, 31.3%) of the respondents sent samples to the laboratory for microbiology testing to guide their choice of antimicrobial agents for therapeutic purposes. Slightly over three-quarters (n=109, 75.7%) possess the veterinary formulary (VF) however, only a hand full of these (n=14, 12.8%) always used the VF when deciding upon the appropriate antimicrobials to use for a patient.

**Fig. 3:**
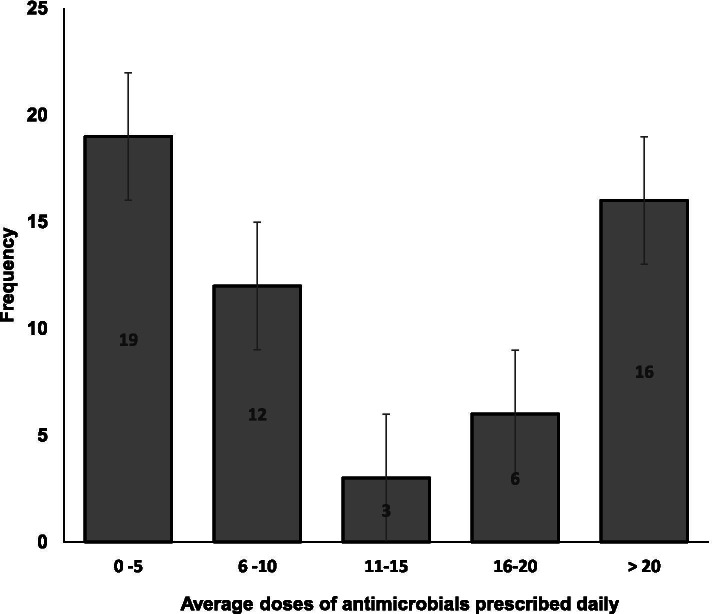
The average doses of antimicrobial agents prescribed daily by Veterinarians in Abuja. The bars represent the distribution of the average frequency of antimicrobial agents prescribed daily by the veterinarians. *Error bars represent Standard Error of the mean

Veterinarians (n=97) who indicated that they prescribed antimicrobials empirically, reported that the three most prescribed antimicrobials were tetracycline (n=91, 93.8%), penicillin (n=42, 43.3%) and gentamicin (n=37, 38.1%). Less than half (n=71, 49.3%) of the respondents declared that they had changed their prescribing behavior in the light of AMR over the past 5 years. Of 97 veterinarians who indicated that they prescribed antimicrobials, only 56 (57.7%) declared the average number of antimicrobials prescribed daily. Most (n=19, 33.9%) declared that they prescribed an average of five (5) antimicrobials, however, (n=16, 28.6%) prescribed over 20 antimicrobials daily (Fig. 3).

Regarding advice provided by the veterinarian to the clients on antimicrobial usage, 128 (88.9%) of the respondents declared that they usually advise that patients must complete the full course of antimicrobials for the recommended duration. Most 113 (78.5%) declared that they insisted that patients must take the antimicrobials at the correct time intervals, 63 (43.8%) declared that they usually advised that the client must dispose of the remaining antimicrobials appropriately, 116 (80.6%) declared that the client must not stop treatment even if there is improvement after a few doses. Most worrisome, 10 (6.9%) of the respondents declared that they usually advised clients to save the remaining antimicrobials for use the next time the animals are unwell.

### Factors influencing antimicrobial prescribing practices of veterinarians

Several factors were analysed to determine if there was any association between those factors and having the right attitude towards antimicrobial prescribing practice. Of all the seven factors considered, only two factors were statistically significant at bivariate analysis (Table 3).

**Table 3:** Bivariate analysis of factors influencing antimicrobial prescribing practices of veterinarians

Factors	Good Attitude	Poor Attitude	Odds Ratio	95% Confidence Intervals	P-value
**Age group (years)**					
< 30 – 39	24	60	1.44	0.66 – 3.14	0.34
> 30 – 39	13	47			
**Gender**					
Female	15	25	2.24	1.01 – 4.94	0.04*
Male	22	82			
**Year of Graduation**					
< 2000	8	24	0.95	0.38 – 2.35	0.92
> 2000	29	83			
**Level of Education**					
D.V.M	16	43	1.13	0.53 – 2.41	0.74
Higher degree	21	64			
**Practice Location**					
AMAC	28	80	1.05	0.44 – 2.50	0.91
Outside AMAC	9	27			
**Field of Practice**					
Government	25	59	1.69	0.77 – 3.72	0.18
Private Sector	12	48			
**Knowledge of AMS**					
Good	32	64	4.30	1.55 – 11.90	0.003*
Poor	5	43			

*Factors that are statistically significant at p *<* *0.05*

The findings of our study showed that being a female veterinarian was an important factor associated with having the right attitude towards antimicrobial prescribing. Female veterinarians were two times more likely to have the correct attitude towards antimicrobial prescribing (Odds Ratio [OR] =2.24; 95% confidence interval [CI] =1.01 – 4.94). Our results show that another important factor influencing the prescribing practices of veterinarians in Abuja was having good knowledge of AMR and AMS (Table 3). Veterinarians who had good knowledge of AMR and AMS were four times more likely to display good antimicrobial prescribing practices (OR = 4.30; 95% CI = 1.55 – 11.90).

### Survey of antimicrobials sold by veterinary drug stores

**Fig. 4:**
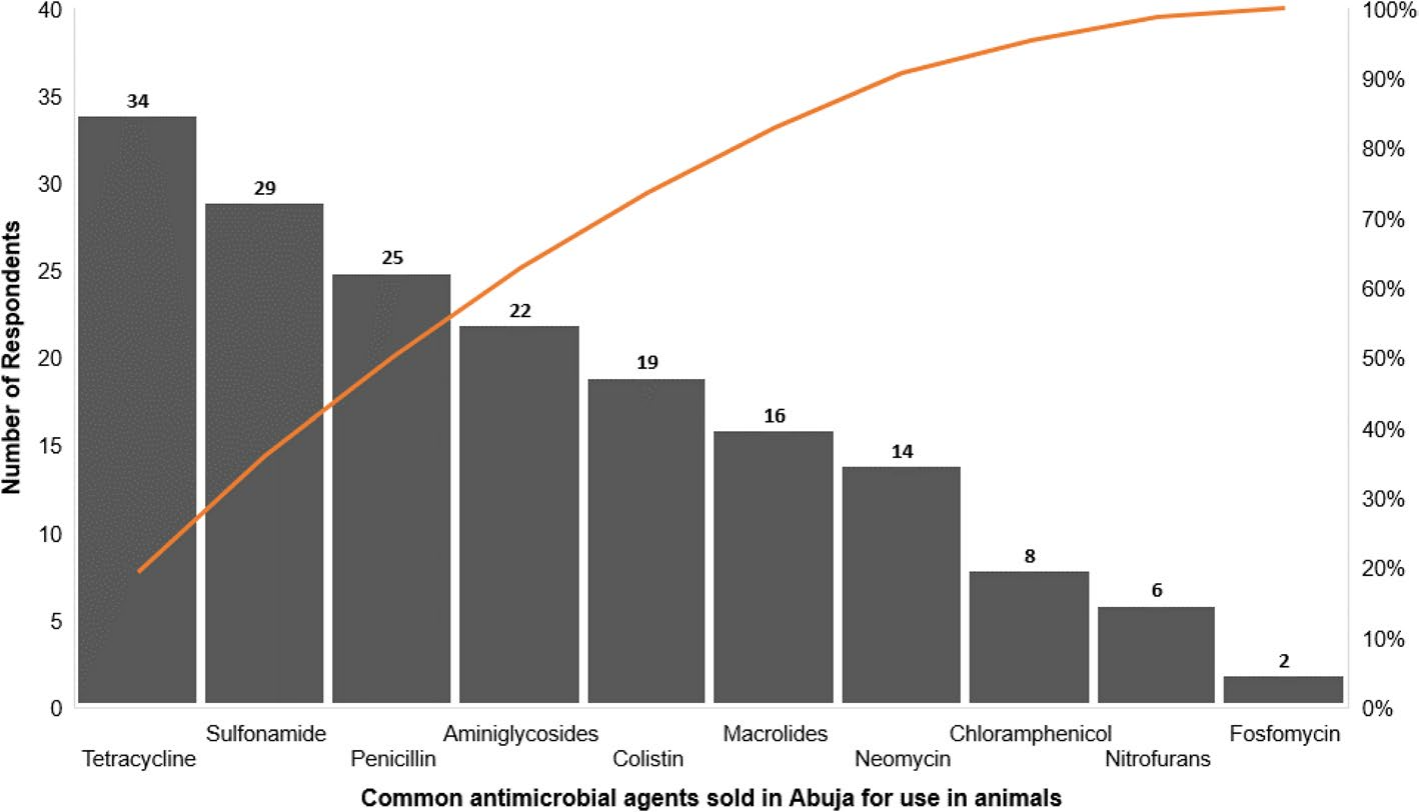
Common antimicrobial agents sold in Abuja by Veterinarians. This chart shows the distribution of frequency of sales of antimicrobials as reported by the respondents in descending order with a cumulative line on the secondary axis as a percentage of the total. The most common antimicrobial agent sold in Abuja by veterinarians for use in animals as declared by the respondents was tetracycline while the least sold was fosfomycin

Of 144 respondents, only 25% (n=36) sold antimicrobial agents. Among those who indicated that they sold antimicrobial agents, 75% (n=27) were retailers while 25% were wholesalers. Figure 4 displays the different antimicrobials sold by veterinary drug stores. The most common antimicrobials sold for use in animals were tetracycline (94.4%, n=34), sulfonamide (80.5%, n=29), penicillin (69.4%, n=25), aminoglycosides (61%, n=22), colistin (52.8%, n=19), neomycin (38.8%, n=14), macrolides (44.4%, n=16), chloramphenicol (22.2%, n=8), nitrofurans (16.7%, n=6) and fosfomycin (5.5%, n=2).

**Fig. 5:**
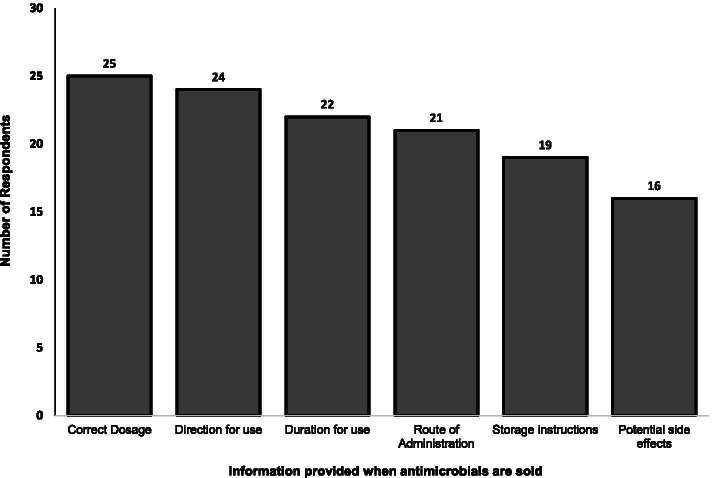
Information provided by veterinarians when antimicrobials are being sold. Each bar represents the distribution of information provided by the veterinarian when the clients purchase antimicrobials

Respondents declared that these antibiotics or classes of antibiotics were usually sold without a written prescription; tetracycline, sulfonamide, penicillin, chloramphenicol, aminoglycosides, cephalosporins, macrolides, colistin, and fosfomycin. Respondents declared that they usually provided clients/farmers with the following information when antimicrobials are being purchased; correct dosage (69.4%, n=25), direction for use (66.7%, n=24), correct duration for use (61.1%, n=22), correct route of administration (58.3%, n=21), storage instructions (52.7%, n=19) and potential side effects (44.4%, n=16) as shown in Fig. 5.

A majority (69.4%, n=25) of the respondents who sold antimicrobial agents in Abuja declared that they would advise the client on the dosage of antimicrobials to give the animals. However, only 44.4% (n=16) of the respondents would advise the client on the potential adverse or side effects of the drugs on the patient.

Majority (94.4%, n=34) of the respondents who sold antimicrobials had poultry farmers as their clients; 72.2 % (n=26) companion animals; 52.8% (n=19) sheep and goat farmers; 36.1%, (n=13) were dairy or beef cattle farmers and 22.2% (n=8) had pig farmers as their clients. Most (44.4%, n=16) of the respondents who sold antimicrobial agents declared that the number of clients/farmers purchasing antimicrobials without prescription daily was less than 20. However, 27.8% (n=10) of the respondents who sold antimicrobial agents declared that the number of clients/farmers purchasing antimicrobials without prescription daily was more than 20 but less than 50. About a quarter of respondents (27.8%, n=10) who also sold antimicrobial agents declared that the number of clients/farmers purchasing antimicrobials without prescription daily was more than 50. Regarding the various sources of antimicrobial agents for sale by veterinarians, the majority (94.4%, n=34) purchased the antimicrobials that they sold from drug distribution companies. More than half (69.4%, n=25) of the respondents who sold antimicrobials sourced their supply from neighboring wholesalers while 52.8% (n=19) sourced their supply from wholesalers in another location in the Federal Capital Territory. More than two-thirds (44.4%, n=16) sourced their supply from wholesalers outside Abuja while 44.4% (n=16) usually get their supply from local drug manufacturing companies. However, only a few (22.2%, n=8) got their supplies from an international source.

## Discussion

This is the first study to assess knowledge, attitudes and practices on antimicrobial stewardship (AMS) among veterinarians in Abuja, Nigeria and to survey the veterinary drugs sold in the study area. Data on AMR and AMS generated from this study is important for the implementation of the AMR NAP. The dangers associated with the abuse and misuse of antimicrobials in animal health cannot be overemphasized as it negatively influences human and animal health. Hence, AMS programmes can help improve the prescribing patterns of veterinarians as well as provide valuable information that can improve patient wellbeing. The present study demonstrated that the overall knowledge of veterinarians regarding AMR and AMS was poor. This inadequate knowledge could be translated to a lack of awareness of AMR and AMS among the study population. Although, majority of the respondents were aware of the causes of AMR including poor stewardship practices such as the use of antimicrobials as growth promoters, non-compliance of farmers with prescribed treatment, overuse of antimicrobials without prescriptions, errors in veterinary prescription (dose, duration of use and choice), clients pressure for antimicrobial prescriptions amongst others.

This is similar to findings of other related studies done in other parts of Nigeria that also reported poor knowledge of AMR due to the low levels of awareness [18]. It was suggested that the low level of knowledge of AMS observed in these studies could be as a result of deficiency of AMR issues in the curricula of Nigerian veterinary medical schools [17, 19]. A related study in Australia reported that one barrier to AMS was pressure from clients to dispense antimicrobials supporting the results of our study [22]. Although a majority of the respondents had declared that, they had heard about AMS, their overall knowledge was poor. The low level of knowledge about AMS in a similar study carried out by Anyanwu *et al.* (2018) was attributed to the fact that very few (17%) of the respondents had heard of AMS. Poor knowledge of AMS observed in our study is rather not surprising, as 52.3% of our respondents declared that they had never attended any training on AMR and stewardship. The majority of our respondents advocated that improving educational campaigns were vital to combatting AMR and this is consistent with the findings of other related studies [17, 18, 23].

Overall, 25.7% of our respondents displayed good attitude/prescribing behaviour towards AMS although some respondents declared that when in doubt they referred to the VF for information on the appropriate use of antimicrobials. This is in agreement with findings of a study done in South-eastern Nigeria [17] but not consistent with findings from another study among veterinary students where only 14% of respondents declared that they would consult the VF and also at variant with another study in Nigeria where only 9.3% of veterinary professionals referred to the VF [18, 24]. Recent studies have reported the need to update the VF regularly for effective AMS in veterinary practice [20]. The present study revealed that only a few practicing veterinarians who prescribed antimicrobials sent samples to diagnostic laboratories for microbiological testing. The majority declared that prescriptions were based on observation and experience. This finding agrees with reports of other studies in related settings [17, 25, 26]. A similar study done in the UK reported that the cost of diagnostic tests was a major barrier to appropriate prescribing among veterinarians [27]. A possible explanation for our findings was most likely due to the presence of very few laboratories as well as the cost of such diagnostic tests.

Considering the potential role of veterinarians in the execution of AMS programmes, it is imperative to know the practices and attitudes of veterinarians towards AMS [28]. This is crucial to understand the behaviour of veterinarians as important stakeholders involved in prescribing and dispensing antimicrobials. It is necessary to determine key areas that will be useful in developing AMS programmes in the veterinary profession and for the education of the general public which is an integral part of all AMR containment activities [15, 23].

Factors positively influencing antimicrobial prescribing practices of veterinarians in our study were gender and good knowledge of AMR and AMS. This is similar to findings of a study done in the UK that reported AMR awareness and professional learning as factors that facilitated the appropriate prescribing practice of veterinarians [27]. However, while there are several reports on knowledge, perception, behavior, and practices of AMS among different healthcare professional such as pharmacists, dentists and doctors globally, there is a paucity of information [28, 29] regarding the perception/level of awareness, attitude, behaviour and practices of AMS among veterinarians in the available literature.

Regarding the survey of antimicrobials sold in Abuja, our results reveal that the most commonly purchased drug was tetracycline and usually sold without a written prescription. This is similar to findings of other studies done in Tanzania, Serbia, and Ghana where antimicrobials were sold without a written prescription [30–33]. Our study observed that the five most commonly purchased antimicrobials were tetracycline (94.4%), sulfonamide (80.5%), penicillin (69.4%), aminoglycosides (61%) and colistin (52.8%). However, this is not consistent with findings from a study done in Kenya that reported penicillin as the most common drug sold by veterinary stores, and a reduced level of sale without a veterinarian’s prescription was observed [34]. The highest volume of the antimicrobials sold was purchased by poultry farmers and this is similar to findings of other studies done in South-western Nigeria [35].

Although there is currently no legislation obligating veterinarians to implement AMS programmes in Nigeria, the veterinary profession must imbibe the appropriate prescribing behaviors. Although trends in antimicrobial prescribing are changing, the pressures on the veterinarian’s practice by several factors such as lack of continuous professional development on AMS may result in slow behavior change.

This study is not without limitations as it was conducted in only one location in Nigeria hence the study results cannot be generalized to the entire country. Nevertheless, some of the issues identified by the present study are of widespread relevance as they are likely to be occurring worldwide in similar study settings hence the findings can be used to inform policy when developing interventions targeted at veterinarians. Secondly, there is a possibility of selection bias as the veterinary register issued by the veterinary council of Nigeria was used to obtain a list of veterinarians in the study area. However some veterinarians who were active on social media with good internet access but not actively practicing in the veterinary industry may have participated in the survey and consistent with the literature [24].

## Conclusion

Overall, having good knowledge and a good attitude regarding AMR and AMS among veterinarians practicing in Abuja were poor. Being a female veterinarian and having good knowledge of AMR and AMS were identified as factors influencing the prescribing practices of veterinarians in Abuja. Even though a higher proportion of respondents demonstrated moderate knowledge about AMR and AMS, this study identified erroneous perception among respondents concerning AMS. Although respondents who had good knowledge of AMR and stewardship were more likely to demonstrate appropriate prescribing behaviors than those with poor knowledge, efforts must be made towards addressing the gap in knowledge among the veterinarians in the study location and the entire country. Most respondents often referred to the VF when in doubt of the appropriate antimicrobial agent to administer. The most common antimicrobial sold without prescription was tetracycline, while the majority of those who purchased antimicrobials from veterinary stores were poultry farmers. We recommend that government should organize awareness campaigns aimed at highlighting the dangers of indiscriminate use of antimicrobials in food animals as well as educate farmers on alternatives to antimicrobials such as vaccination, biosecurity measures, probiotics, etc hence reducing the overdependence on antimicrobials. The VF should be updated following the WHO list of critically-important-antimicrobials (CIA) and veterinarians should be educated not to use these CIAs in the treatment of food-producing animals.

## Supplementary Information

**Additional file 1.**

**Additional file 2.**

## Data Availability

The datasets used and analyzed during the current study are available from the corresponding author on reasonable request. All data generated or analyzed during this study are also included in this published article [and its supplementary information files].
