# Supplementary material for: Assessing knowledge, attitude, and practices of veterinarians towards antimicrobial use and stewardship as drivers of inappropriate use in Abuja, Nigeria
Source: One Health Outlook. 2021 Dec 20;3:25. doi: 10.1186/s42522-021-00058-3 (PMC8690525; doi:10.1186/s42522-021-00058-3)
Supplement: Supplementary file 2 — Additional file 2. [file 42522_2021_58_MOESM2_ESM.pdf]

Appendix 1

Assessing Knowledge, Attitude and Practices of Veterinarians towards Antimicrobial Use and Stewardship as drivers of inappropriate use

SELF-ADMINISTERED QUESTIONNAIRE

Introduction and consent section:

INFORMED CONSENT

Dear Colleague,

We kindly request your participation in a survey as a prescribing or dispensing veterinary professional. The aim is to assess the knowledge, attitudes and practices (KAP) of veterinarians on antimicrobial resistance and antimicrobial stewardship in Abuja, Nigeria.

Antimicrobial resistance (AMR) a growing threat in both the developing and developed countries causing problems in all spheres of human health, animal health, the food industry and agriculture. Your participation in this study involves answering questions about antimicrobial resistance and antimicrobial stewardship. The questions will be open-ended and close-ended and will take approximately 20 minutes. Your participation is entirely voluntary. There are no known risks in participating in this study as confidentiality and anonymity is guaranteed by the electronic survey format. Your participation is critical for the success of this research study and entirely voluntary, you are free to decline from participation. If you wish to proceed after reading this letter, you are giving voluntary informed consent by proceeding with the survey.

Thanking you in advance for your participation. If you have any questions please feel free to contact the following: Researcher: **Dr. Mabel Aworh on** mabelaworh@yahoo.com **or +2348032377831**

SECTION 1: DEMOGRAPHIC INFORMATION

1. **What is your gender?**

   Please choose only one of the following:

   a) Male
   b) Female
   c) Do not wish to disclose

2. **Which age group do you fall under?**

   a) 20 – 29 yrs
   b) 30 – 39 yrs
   c) 40 – 49 yrs
   d) 50 – 59 yrs
   e) 60 and above

3. **In what year did you graduate?**

4. **At which University did you obtain your DVM?**

5. **What is your highest qualification?**

   a) DVM
   b) MSc
   c) PhD
   d) MCVSN
   e) FCVSN
   f) Others pls specify**……………..**

6. **What is your field of practice?**

   a) Government
   b) Academia
   c) Private practice
   d) Veterinary Pharmacy
   e) Others please specify………………..

7. **Which Area Council in FCT do you practice?**

a) AMAC
b) Kuje
c) Kwali
d) Bwari
e) Gwagwalada
f) Abaji

SECTION 2: KNOWLEDGE

8. Antimicrobial resistance as a serious problem in Nigeria, I
a) Strongly Agree
b) Agree
c) Neutral
d) Disagree
e) Strongly Disagree

9. How confident are you on your knowledge of antimicrobials, antimicrobial resistance and stewardship? (Rate yourself on a scale of 1 to 5 with 1 being least confident and 5 being very confident)
a) Antimicrobials☐ 1 ☐ 2 ☐ 3 ☐ 4 ☐ 5
b) Antimicrobial resistance☐ 1 ☐ 2 ☐ 3 ☐ 4 ☐ 5
c) Antimicrobial stewardship☐ 1 ☐ 2 ☐ 3 ☐ 4 ☐ 5

10. Are antimicrobial drugs effective in treating acute viral infections?

☐ Yes ☐ No ☐ Don't know

11. Please select the oneyou believe to be the most contributory towards antimicrobial resistance? Choose all that apply:
a) Overuse of antimicrobials by prescriptions
b) Overuse of antimicrobials without prescriptions
c) Errors in veterinary prescriptions (dose, duration of use and choice)
d) Non-compliance of clients/farmers with the prescribed treatment
e) Inadequate hygiene and biosecurity measures
f) Lack of vaccinations
g) Lack of new antimicrobial drugs
h) Use of antimicrobials as growth promoters in animals
i) Clients/Farmers pressure for antimicrobial prescriptions
j) Others please specify …….

12. **Which of the following sources do you refer to for information on appropriate usage of antimicrobials?** Please select your appropriate choice and rate on a scale of 1-5 (1=lowest preference and 5=highest preference)

Please choose the appropriate response for each item

a) Information from senior colleagues ☐ 1 ☐ 2 ☐ 3 ☐ 4 ☐ 5
b) Veterinary Formulary ☐ 1 ☐ 2 ☐ 3 ☐ 4 ☐ 5
c) Merck Manual ☐ 1 ☐ 2 ☐ 3 ☐ 4 ☐ 5
d) Veterinary Journals ☐ 1 ☐ 2 ☐ 3 ☐ 4 ☐ 5
e) OIE Guidelines ☐ 1 ☐ 2 ☐ 3 ☐ 4 ☐ 5
f) Veterinary websites ☐ 1 ☐ 2 ☐ 3 ☐ 4 ☐ 5

13. **Please assess your levels of confidence in the following areas when prescribing antimicrobials?** (1=very confident, 2=confident, 3=Unsure, 4= Unconfident, 5=very unconfident, 6=Not Applicable)
Please choose the appropriate response for each item

a) Making an accurate diagnosis of the infection ☐ 1 ☐ 2 ☐ 3 ☐ 4 ☐ 5 ☐ 6
b) Decision not to prescribe antimicrobials
when not sure of diagnosis ☐ 1 ☐ 2 ☐ 3 ☐ 4 ☐ 5 ☐ 6
c) Selecting the correct antimicrobial ☐ 1 ☐ 2 ☐ 3 ☐ 4 ☐ 5 ☐ 6
d) Selecting the correct dosage of antimicrobial ☐ 1 ☐ 2 ☐ 3 ☐ 4 ☐ 5 ☐ 6
e) Selecting the correct duration for antimicrobial ☐ 1 ☐ 2 ☐ 3 ☐ 4 ☐ 5 ☐ 6
f) When to stop the antimicrobial agent ☐ 1 ☐ 2 ☐ 3 ☐ 4 ☐ 5 ☐ 6

14. Do you think there are risks associated with irrational use of antimicrobials?
☐ Yes ☐ No ☐ Don't know

15. If you answered yes to the previous question, please list the risks
……………………………………………………………………………………………
……………………………………………………………………………………………
…………

16. Do you think restricting antimicrobial usage in veterinary care is necessary to reduce antimicrobial resistance?
☐ Yes ☐ No ☐ Don't know

17. Do you believe a patient skipping /missing one or two doses of antimicrobials contributes to the development of antimicrobial resistance?
☐ Yes ☐ No ☐ Don't know

18. Are there possibilities that new classes of antimicrobials will be developed in the next 5 to 10 years?
☐ Yes ☐ No ☐ Don't know

19. Are you aware that you may be contributing towards antimicrobial resistance in your veterinary practice?
☐ Yes ☐ No ☐ Don't know

20. Have you attended any workshops or had training on antimicrobials and antimicrobial stewardship?

☐ Yes ☐ No ☐ May be

21. If you selected Yes on the previous question, kindly state when you attended the workshops or training.

22. Would you like more education and training on antimicrobial use, antimicrobial resistance and antimicrobial stewardship?

☐ Yes ☐ No ☐ Don't know

23. Please list the three most resistant bacteria you are aware of?

…………………………………………………………………………….

24. What percentage of clinical antimicrobial use is unnecessary in Nigeria?

a) Between 1 – 20%
b) 21 - 40%
c) 41 – 60%
d) 61 – 80%
e) 81 – 90%

☐ Don't know

25. What percentage of clinical antimicrobial use is inappropriate in Nigeria?

a) 1 – 20%
b) 21 - 40%
c) 41 – 60%
d) 61 – 80%
e) 81 – 90%

☐ Don't know

SECTION 3: ATTITUDES

26. Antimicrobials are safe drugs that can be commonly prescribed?

☐ Yes ☐ No ☐ Don't know

27. Prescribing antimicrobials to healthy animals as a form of prophylaxis may ultimately have negative impact on their health, I

a) Strongly Agree
b) Agree
c) Neutral
d) Disagree
e) Strongly Disagree

28. In your opinion do you think the problem of antimicrobial resistance is getting better or worse?

☐ Better ☐ Worse ☐ Don't know

29. What do you think are the important strategies to combat antimicrobial resistance? Select the appropriate. Choose all that Apply

a) Educational campaigns
b) Use of treatment guidelines
c) Vaccination campaigns
d) Improved biosecurity in farms, clinics, hospitals
e) Reducing antimicrobial use in agriculture and animals
f) Better control of antimicrobial sales
g) Others, please specify…………………………………………….

SECTION 4: PRACTICE

30. Do you possess the annual veterinary practicing license issued by the Veterinary Council of Nigeria?

☐ Yes ☐ No

31. Do you prescribe antimicrobial drugs to your clients?

☐ Yes ☐ No ☐ Not Applicable

32. Please select the average number of patients you prescribe antimicrobials per day?

33. Do you prescribe antimicrobials empirically? (based on observation and experience)

☐ Yes ☐ No ☐ Not Applicable

34. Before initiating antimicrobial therapy do you send samples for microbiology testing to inform the need for and/or choice of antimicrobial therapy?

☐ Yes ☐ No ☐ Not Applicable

35. Do you possess the latest Nigerian Veterinary Formulary?

☐ Yes ☐ No

36. How often do you use the Nigerian Veterinary Formulary or Treatment Guidelines when deciding upon antimicrobials to use for a patient?

☐ Always

☐ Sometimes

☐ Never

☐ Not Applicable

37. Which three (3) antimicrobials do you prescribe the most?

…………………………………………………………………………….

38. Have you changed your prescribing behavior in the light of antimicrobial resistance in the past 5 years?

☐ Yes ☐ No ☐ Don't know

39. If yes, comment on How

……………………………………………………………………………………………
……………………………………………………………………………………………
……………………………………………………………………………………………
…………………………………………………………………………………

40. What advice do you impart to your clients on antimicrobial usage? Choose all that Apply

☐ Patient must complete the full course of antimicrobials for the recommended duration
☐ Patient must take the antimicrobials at the correct time intervals

☐ Patient must dispose of remaining antimicrobials appropriately

☐ Patient must not stop treatment even if there is improvement after a few doses

☐ Patient must save remaining antimicrobials for the next time they are unwell

SECTION 5: SURVEY ON SALE OF ANTIMICROBIALS

41. Do you sell antimicrobial agents? ☐ Yes ☐ No

42. If yes, are you a retailer or wholesaler?

☐ Retailer

☐ Wholesaler

☐ Both

43. If yes, what classes of antimicrobial agents do you sell? Select all that applies

☐ Sulfonamides

☐ Tetracycline

☐ Pencillin

☐ Macrolides

☐ Fosfomycin

☐ Chloramphenicol

☐ Aminoglycosides

☐ Colistin

☐ Cephalosporins

☐ Others please specify…………………………………………………

44. What are the Sources of antimicrobial agents for sale? Select all that applies

☐ Neighboring wholesaler

☐ Wholesaler in another location in FCT

☐ Wholesaler outside Abuja

☐ Drug distribution company

☐ Drug manufacturing company

☐ Import from an international source

☐ Others please specify………………………………………………………

45. Number of clients/farmers purchasing antimicrobial agents without prescription daily?

☐ less than 20

☐ greater than 20 but less than 50

☐ greater than 50

46. What are the classes of antimicrobial agents sold without needing a written prescription?...

☐ Sulfonamides

☐ Tetracycline

☐ Pencillin

☐ Macrolides

☐ Fosfomycin

☐ Chloramphenicol

☐ Aminoglycosides

☐ Colistin

☐ Cephalosporins

☐ Others please specify………………………………………………..

47. What are the commonly sold antimicrobials to different types of farmers/clients? Please tick all that applies

☐ Sulfonamides

☐ Tetracyclines

- ☐ Pencillins
- ☐ Neomycin
- ☐ Nitrofurans
- ☐ Chloramphenicol
- ☐ Erythromycin
- ☐ Colistin
- ☐ Cephalosporins
- ☐ Others please specify……………………………………….

48. What are the information you provide to a client/farmer when purchasing antimicrobials? Tick all that applies

- ☐ Correct dosage
- ☐ Directions for use
- ☐ Storage instructions
- ☐ Potential side effects
- ☐ Correct route of administration
- ☐ Correct duration of use

49. Who are the main clients

- ☐ Poultry farmers
- ☐ Dairy/Beef farmers
- ☐ Pig farmers
- ☐ Sheep and goat farmers
- ☐ Companion animal owners (dogs/cats)
- ☐ Others, please specify …………………………………………….

50. On the average, what quantity of antimicrobials do you sell daily?

Thank you for your time
