# Supplementary material for: Assessing knowledge, attitude, and practices of veterinarians towards antimicrobial use and stewardship as drivers of inappropriate use in Abuja, Nigeria
Source: One Health Outlook. 2021 Dec 20;3:25. doi: 10.1186/s42522-021-00058-3 (PMC8690525; doi:10.1186/s42522-021-00058-3)
Supplement: Supplementary file 1 — Additional file 1. [file 42522_2021_58_MOESM1_ESM.docx]

15^th^ August 2021

**The Editor,**

**BMC One Health Outlook Journal**

Dear Editor,

The manuscript entitled: “**Assessing Knowledge, Attitude, and Practices of Veterinarians towards Antimicrobial Use and Stewardship as drivers of inappropriate use**” was written by myself and co-authors, Jacob Kwaga, and Emmanuel Okolocha.

This original work has not been published or been considered for publication elsewhere. I hereby submit the manuscript on behalf of my co-authors for consideration for publication in the BMC One Health Outlook Journal.

We are certain that this manuscript will be useful and of great interest to your readership of public health policy makers, clinicians and researchers in Antimicrobial Resistance prevention because it addresses the knowledge, attitude and practices towards antimicrobial use and stewarship in a unique study population: veterinarians. The rationale for this study is to provide base line data for the implementation of the AMR National Action Plan in Nigeria using a One Health Approach.

All the authors participated in this study, writing the manuscript and consented to the submission of the manuscript.

Should you require additional information or if you have any questions, please contact me. I will be pleased to hear from you.

Yours sincerely,

**Dr. Mabel Aworh (**Corresponding author)

D.V.M, MPH

Graduate, Nigeria Field Epidemiology and Laboratory Training Programme,

50 Haile Selassie Street, Abuja, Nigeria.

mabelaworh@yahoo.com, +234 -8032377831
